# Structured Lifestyle Modification Prior to Bariatric Surgery: How Much is Enough?

**DOI:** 10.1007/s11695-021-05573-w

**Published:** 2021-07-23

**Authors:** John Brazil, Francis Finucane

**Affiliations:** 1 Bariatric Medicine Service, Centre for Diabetes, Endocrinology and Metabolism, Galway University Hospitals, Saolta Health Care Group, Galway, Ireland; 2 HRB Clinical Research Facility, NUIG and Galway University Hospitals, Saolta University Health Care Group, Galway, Ireland; 3 Department of Medicine, School of Medicine, CMNHS, NUI Galway, Galway, Ireland

**Keywords:** Obesity, Bariatric surgery, Structured lifestyle modification

## Abstract

Many healthcare systems require patients to participate in a structured lifestyle modification programme prior to bariatric surgery, even though bariatric consensus guidelines do not recommend this. While there is good evidence that such programmes improve health in other conditions such as metabolic and cardiovascular diseases, there is no evidence that they improve outcomes after bariatric surgery. The distinction needs to be drawn between the well-established need for individualised multidisciplinary dietetic and physical activity care for bariatric surgical patients and the potential harms from mandating participation in compulsory structured lifestyle programmes of fixed duration, frequency and intensity, which may delay surgery, reinforce obesity stigma, or both. Large clinical trials might help to address some of the uncertainty and provide an evidence base for clinicians and policymakers.

**Graphical abstract:**

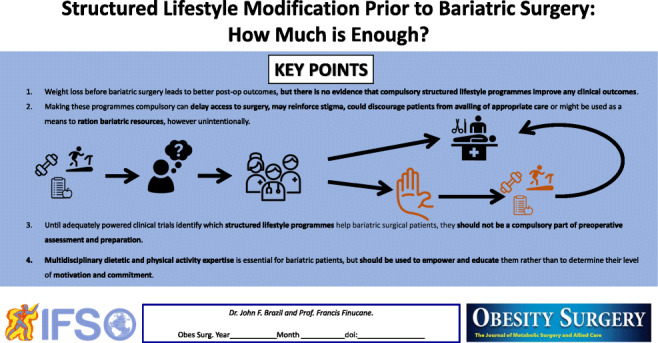

## Introduction

The prevalence of severe obesity continues to rise [1] and bariatric surgery remains the most effective intervention to reduce mortality [2], morbidity [3] and healthcare costs [4] for affected individuals. While lifestyle modification is always the cornerstone of the therapeutic approach to obesity, the specific role of structured lifestyle modification programmes for patients who are preparing for bariatric surgery remains unclear. On the one hand, the requirement for multidisciplinary specialist input from dietetic and physical activity experts is well established in clinical practice guidelines [5, 6], but on the other, patients are sometimes subjected to an arbitrary requirement to undergo participation in structured lifestyle modification programmes prior to being ‘approved’ for surgery. This is an area of controversy and uncertainty in bariatric practice that has been addressed before [7] but remains as relevant now as ever. In fact, mandating participation in structured lifestyle programmes before surgery actually goes against expert consensus pre-operative recommendations. The *‘Interdisciplinary European Guidelines on Metabolic and Bariatric Surgery 2013*’ [8] advise that patients undergo routine pre-operative assessment as with any other major abdominal surgery including specialist surgical, anaesthetic and dietician input and receive an ‘explanation of the dietary changes required after surgery’ as well as an ‘assessment of patient motivation and willingness to adhere to follow-up programmes’. However, there is no recommendation within the guidelines that this takes the form of a structured, time-based lifestyle intervention programme. More recently, the ‘*Clinical Practice Guidelines For The Perioperative Nutrition, Metabolic, and Nonsurgical Support of Patients Undergoing Bariatric Procedures – 2019 Update: Cosponsored By American Association of Clinical Endocrinologists/American College of Endocrinology, The Obesity Society, American Society For Metabolic & Bariatric Surgery, Obesity Medicine Association, and American Society of Anaesthesiologists*’ [9] make no specific recommendation for participation in a structured lifestyle modification programme before surgery.

Of course, optimising lifestyle behaviours prior to surgery is both desirable and sensible, but whether this is best facilitated by specific members of the bariatric multidisciplinary team tailoring care for each unique patient’s needs, rather than using a pre-defined intervention of fixed structure, duration, frequency and intensity, remains unclear in some care pathways. We sought to explore the evidence base for mandatory participation in structured lifestyle modification programmes before bariatric surgery, to identify any areas of uncertainty and to consider the implications for policymakers and those responsible for structuring care pathways for patients with severe obesity.

## Does Weight Loss Before Bariatric Surgery Improve Outcomes?

Excess body weight is an established risk factor for certain perioperative morbidities in general surgery patients, including wound infections [10] and deep venous thrombosis [11] and it seems reasonable to infer that pre-operative weight loss might reduce these complications. A large single-centre retrospective cohort study in the USA in 2009 found that the magnitude of weight loss in patients undergoing a 6-month pre-operative weight loss intervention (with a target loss of 10%) was inversely proportional to complication rate [12]. A similar study in Finland found those losing more than 10% body weight before gastric bypass surgery had reduced operative time, length of stay and morbidity and had more weight lost at 12 months compared to those who lost less than 5% body weight pre-operatively [13]. A large single-centre cohort study in Poland found that patients losing 5% body weight before surgery lost more weight subsequently than those who lost less than 5% [14]. A Japanese study noted similar findings but was not powered to detect changes in post-operative complications with pre-operative weight loss [15]. Much larger cohort studies, such as the Metabolic and Bariatric Surgery Accreditation and Quality Improvement Program (MSAQIBP) which enrolled patients from the USA and Canada, have demonstrated convincingly that 30-day mortality in bariatric surgical patients is inversely proportional to body mass index (BMI) as well as the magnitude of weight loss prior to surgery [16]. Interestingly, however, the same analysis by a different group of authors of the same cohort 1 year previously had found no such associations, but rather an increased risk of surgical site and urinary tract infections in those with lower BMI [17]. This highlights the inherent limitations of even very large and well-conducted retrospective cohort studies. In order to allow higher level causal inference and the robust development of sound bariatric clinical care pathways, randomised controlled trials and meta-analyses are required.

## What Clinical Trial Evidence Supports Mandatory Participation in a Lifestyle Modification Programme Prior to Bariatric Surgery?

The first randomised controlled clinical trial of lifestyle modification to improve post-operative outcomes in bariatric surgical patients was not conducted until 2007 and found no difference in patients who were randomised to a weight loss requirement of 10% versus no such requirement [18]. However, there were just 100 trial participants, 39 of whom were lost to follow-up, so the risks of bias and confounding, as well as being underpowered to detect an intervention effect, were high. Even though a similar trial at the time found that pre-operative weight loss ‘should be encouraged’ because it predicted post-operative weight loss, the allocation to the weight loss intervention (with a mean reduction in excess body weight of 9.3% more than control participants) did not lead to any between-group differences in outcomes [19]. In other words, there was a distinction between weight loss ‘working’, which it did, and being allocated to a weight loss group, which did not have an effect. A larger multicentre trial found that pre-operative weight loss with a 14-day very low-calorie diet (VLCD) did lead to important benefits, including reduced perceived difficulty by the surgeon during the operation and reduced early post-operative complications, but there were no differences in intraoperative complications, blood loss or operative time [20]. This latter trial was distinct from others in that it sought short-term calorie restriction in the immediate pre-operative period in order to achieve a reduction in the size of the liver rather than weight loss per se, in order to make the operations technically less challenging for the surgeon. This highlights the large degree of heterogeneity in this domain of clinical research, such as variations in the lifestyle interventions, their duration and the identification of the most relevant endpoints.

Methodological inconsistencies [21] and the limited number and quality of prospective studies in this area [22] are well-described barriers to the effective establishment of rigorous standards for pre-bariatric surgical care pathways [23]. Where good quality trials have been done, the results have not demonstrated any benefit from mandatory structured lifestyle modification before surgery: In the largest of these trials, the only difference for patients who completed a 6-month lifestyle intervention before surgery compared to controls was that, somewhat unexpectedly, they lost less rather than more weight after 2 years [24]. These findings coincided with the publication of a large retrospective analysis from the USA, showing that insurance company-mandated ‘medical weight management’ prior to bariatric surgery conferred no benefit to patients [25]. Arguably the most authoritative assessment of the influence of lifestyle changes for pre-operative weight loss on surgical outcomes from Roman et al. [26] examined data from 6060 patients across four clinical trials and 12 cohort studies in a meta-analysis. They noted that although these interventions are effective at reducing weight (by an average of 7.4 kg), there was no difference in perioperative morbidity or mortality except for a reduced length of hospital stay by 27%. In considering why the evidence for pre-operative structured lifestyle modification is so poor, it may be that the incidence rates for morbidity and mortality are too low to allow the trials to be adequately powered to detect a between-group difference. Furthermore, ‘per protocol’ rather than ‘intention to treat’ analyses may introduce bias. For example, if patients randomised to control rather than lifestyle interventions did worse but were also more likely to drop out, lifestyle benefits might be obscured. Much larger multicentre trials reporting intention to treat analyses could address these limitations. For now, the objective scientific conclusion must be that higher level evidence from randomised controlled trials and meta-analyses for any benefit from mandatory structured lifestyle modification before bariatric surgery simply does not exist.

## Isn’t the Broader Evidence for Structured Lifestyle Modification in Patients with Severe Obesity Well Established?

It is established beyond doubt that dietary [27] and physical activity [28] behaviours, driven at a population level by complex environmental factors [29, 30], ultimately give rise to excess body weight, and as such, any treatment for obesity addresses this accumulation of excess energy through ‘lifestyle modification’ in some way. Moreover, several large, methodologically robust randomised controlled trials have established the benefits of participation in structured lifestyle modification programmes in distinct patient groups, including those with cardiovascular disease [31], pre-diabetes [32] or established type 2 diabetes [33, 34]. Regarded by many as the most rigorously conducted structured lifestyle intervention trial ever undertaken, Look AHEAD [35] (Action for Health in Diabetes) is particularly relevant to considerations about how best to help patients with severe and complicated obesity through changes in behaviour. The trial recruited more than 5000 adults who were overweight (mean BMI 36 kg m^−2^) with type 2 diabetes from 16 centres in the USA and followed them for 10 years, randomising them to a ‘usual care’ diabetes structured education programme versus an ‘intensive lifestyle intervention’, with an ambitious individual weight loss target of 10%, including meal replacement, if necessary, in the intervention group. Compared to controls, those in the intensive lifestyle intervention lost more weight and had improvements in many important health related outcomes such as diabetes control and medication usage [36], but the primary trial outcome of major adverse cardiovascular events was no different, even after extended follow-up. This may have been due at least in part to the much lower than expected cardiovascular event rate in the control group, and it would be incorrect to dismiss the overwhelming benefits demonstrated by the trial, but it ought not to constitute grounds for mandating participation in such a programme for all overweight patients, even just those with diabetes. It is worth noting that a post hoc analysis of Look AHEAD participants found that those who had bariatric surgery lost on average 19.3% of their body weight compared to 5.6% in the intensive intervention group and 3.3% in the control group and were almost seven times more likely to achieve diabetes remission [37]. Of course, the trial was not designed to determine the relative efficacy of surgery versus lifestyle, or whether lifestyle intervention before surgery is beneficial, but these findings are consistent with the observations from other trials of proven superior efficacy of bariatric surgery over lifestyle modification alone [4].

Moreover, we know from ‘real world’ clinical studies that drop-out from intensive lifestyle interventions tends to be high [38], weight loss is often modest [39], sustained reductions over time are difficult to maintain [40] and while 10% weight loss is generally regarded as a meaningful level with which to improve health [34, 41], this is rarely achieved: In one large general practice-based cohort of UK adults with severe obesity, the annual probability of achieving 5% weight loss was one in eight for men and one in seven for women [42]. Patients seeking clinical care for severe obesity would rather lifestyle modification alone over surgery if the two were equally efficacious [43] and only a minority of patients who fulfil the clinical criteria will choose to have bariatric surgery [44], but mandating participation in a structured lifestyle modification programme before surgery lacks any evidence base and is problematic for several reasons.

## What Potential Harms Could Arise from Making Participation in a Structured Lifestyle Modification Programme Mandatory Before Bariatric Surgery?

While individualised lifestyle advice to patients prior to surgery is sensible and desirable and consistent with guidelines, compelling patients to complete a structured lifestyle programme with pre-defined content and duration poses several potential problems. Firstly, even though the majority of medical insurance providers in the USA still require a supervised medical weight management programme prior to approval for bariatric surgery, the evidence for this requirement is weak [45]. These requirements have been associated with a lower likelihood of progression to surgery and an increase in the time that patients have to wait for surgery [46]. One study showed a 50% increase in the drop-out rate prior to surgery in patients who had to undergo a 13-week pre-operative dietary counselling programme compared to patients with no such requirement, with no subsequent difference in their outcomes after surgery [47]. Another study showed a threefold increase in mortality where delays occurred compared to where surgery was provided in a timely fashion [48].

Recent iterations of international consensus guidelines have emphasised unequivocally and emphatically that participation in structured lifestyle modification to achieve weight loss prior to surgery should not be mandatory ‘since a likely adverse effect of failure [to lose weight] is denial of a potentially life-saving procedure’ [5]. Moreover, mandatory lifestyle participation tends to feature in publicly funded care pathways rather than in those in the private sector. This has the potential to aggravate socioeconomic disparities in access to bariatric surgery that are already well established and problematic [49]. For example, in Ireland, where the introduction of a ‘tiered’ bariatric care pathway incorporating mandatory lifestyle modification programmes is being considered, there is a high prevalence of severe obesity, with 7.4% of adults over the age of 50 years fulfilling criteria for bariatric surgery [50], but with fewer than 1 per 100000 people receiving surgery, in contrast to rates of 70 per 100000 in Sweden and France and 50 per 100000 in the USA [51]. Often, access to publicly funded structured lifestyle programmes is itself very limited, which acts as a further constraint to access to surgery for affected patients. A perverse incentive may exist for funders to configure pathways in this way in order to curtail resource utilisation, but this would pose a breach of the fundamental principles of medical ethics as they pertain to autonomy, justice, beneficence and non-maleficence for bariatric patients [52]. Ultimately, while individualised lifestyle assessment and education from the bariatric MDT is a core component of care, insisting that patients participate in structured lifestyle modification before surgery where no evidence for its benefit exists could reinforce the pejorative view held by many in society, including doctors [53] and nurses [54] that obesity is a manifestation of a lack of discipline or adequate effort. It could also give rise to patients avoiding bariatric care, compounding low self-esteem and embarrassment [55] and worsening their eventual outcomes [56].

## What Are the Implications for Policies to Deliver Bariatric Care for Patients with Severe Obesity?

It is clear that as a barrier to surgery or as a trial to test motivation or adherence, lifestyle intervention programmes should not be used to determine suitability for bariatric surgery and can cause a potentially harmful delay. While it is entirely reasonable to explore dietary and physical activity behaviours with patients who have excess body weight, structured lifestyle programmes should be seen as educational and empowering for patients, not merely therapeutic in their own right. To draw an analogy, we know that excess body weight is the single most important factor for developing symptomatic gallstone disease [57], but it would be considered ludicrous to introduce mandatory lifestyle modification as a prerequisite for (or a therapeutic alternative to) laparoscopic cholecystectomy. Some lifestyle programmes such as Look AHEAD have proven efficacy in their own right, but surgery has been shown to have far superior efficacy in patients who choose and need it [58]. As the ASMBS stated 5 years ago ‘the discriminatory, arbitrary, and scientifically unfounded practice of insurance-mandated pre-operative weight loss contributes to patient attrition, causes unnecessary delay of lifesaving treatment, leads to the progression of life-threatening co-morbid conditions, is unethical, and should be abandoned’ [59].

It could be argued that the absence of evidence of benefit from pre-operative lifestyle modification programmes does not constitute ‘evidence of absence’ of benefits and that these programmes should be mandated on the basis that they are likely to do some good. Indeed, the significant heterogeneity in the intensity, duration, frequency of contact and mode of delivery of these programmes may account for some of the difficulty in meta-analysing their potential clinical effects. But this approach does not take into account the potential harms caused by delayed care, drop-out from surgery and reinforcement of stigma. At the very least, while individualised bariatric team input for each unique patient to facilitate achievement of healthier lifestyle patterns is sensible and desirable, structured lifestyle modification before surgery needs to be recognised as an uncertain strategy with potential benefits and harms that needs to be objectively and rigorously assessed in large multicentre randomised controlled trials with meaningful clinical endpoints such as metabolic and cardiovascular morbidity and mortality. The distinction should be drawn between short-term pre-operative calorie restriction to reduce hepatic congestion [60] and longer-term pre-operative behavioural interventions mandated to achieve quasi-protective weight loss and act as an indicator of motivation and suitability for surgery.

Unquestionably, patients need multidisciplinary input from dieticians, psychologists and bariatric specialists to help them prepare for a life altering procedure which requires long-term follow-up [61], but this should be a period of individual patient education and preparation rather than being programmatic and tied to the ‘achievement’ of weight loss. It is important to recognise the need for some sort of assessment criteria for suitability for bariatric surgery. Our own clinical practice would be to adhere to internationally recognised thresholds for BMI in the presence or absence of co-morbidities, noting the limitations of BMI. So for example, we do not currently offer surgery to patients with a BMI under 35 kg m^−2^, even where co-morbidities such as type 2 diabetes are present. Next, patients must undergo an assessment with the specialist bariatric psychologist, though we would see this as a way of identifying potential contraindications such as uncontrolled binge eating disorder or undisclosed suicidality as well as making patients aware of the potential psychological issues that arise after surgery (and available supports for those). We do not see the screening psychological assessment as an opportunity to assess ‘motivation’ or willingness to change. We also consider attendance at clinic and engagement with the MDT an important indication of suitability for surgery, but we are very mindful of confounding factors such as lengthy waiting lists and journey times for our patients. Lastly, an assessment by our dietitian is critically important, as it allows individualised, expert dietetic advice to be provided to patients both in terms of quantifying their baseline dietary patterns and determining what specific changes may suit the patient best, as well as providing guidance on post-operative nutritional issues. We do not consider weight loss to be an important indicator of motivation or suitability for surgery in these patients. Again, our own clinical practice is to make available to patients a 10-week structured lifestyle modification programme with nursing, dietetic and physical activity expertise [39] but this is not necessarily a compulsory component of the assessment of whether or not a patient should proceed to surgery, especially when the patient has previously completed a structured programme elsewhere previously.

The focus of the multidisciplinary team should not be to try to motivate the patient to lead a more responsible lifestyle, rather it should be to empower them and ensure the nutritional adequacy of their diet as well as their psychological and physical well-being after surgery. That is not to diminish the responsibility and the duty of self-care that each patient has for their own health, as with any disease or illness. Where an obesity treatment is successful at changing the patient’s underlying physiology to bring about improved regulation of appetite, it is an established ethical principle that they should take responsibility to adhere to that treatment, be it drug therapy or a specific form of lifestyle modification [62, 63]. Conversely, health care professionals have a responsibility to understand that rather than reflecting adequate motivation or self-control, a good response to lifestyle modification reflects favourable biological effects on subcortical areas of the brain that are independent of patients’ agency or discretion [64].

It may be that in certain situations, such as in the presence of an exceptionally high BMI (say, above 70 kg m^−2^) where perioperative morbidity and mortality is particularly high [65], a minimum amount of weight loss ought to be mandated prior to surgery — but we do not yet know what that threshold is and an individualised approach, with multidisciplinary team consensus, should be taken with each patient pending those scientific discoveries. In the meantime, those developing contemporary, evidence-based bariatric care pathways ought to recognise the lack of evidence for mandated structured lifestyle modification before surgery. Medical research funders should prioritise large-scale multicentre randomised controlled trials to address some of the uncertainty in this area and identify which programmes, if any, are suitable for which patients. As in other domains of clinical activity [66], the distinction needs to be drawn between evidence-based and eminence-based practice and we need to be less slow to abandon therapies that provide little or no benefit to our patients.
